# In Vitro Modeling of Tendon with Type I Collagen Scaffolds

**DOI:** 10.3390/gels12100871

**Published:** 2026-09-26

**Authors:** Katherine M. Arnold, Tannin Schmidt, Alix Deymier

**Affiliations:** Department of Biomedical Engineering, UConn Health, Farmington, CT 06030, USAdeymier@uchc.edu (A.D.)

**Keywords:** tendon, collagen, scaffold, crosslinking, genipin

## Abstract

In vitro collagen scaffolds provide simplified systems for investigating tendon properties without the use of animal models. However, common scaffold processing approaches, including dehydration and chemical crosslinking, can alter scaffold water content, molecular structure, thermal, and mechanical properties in ways that are not fully understood. In this study, we investigate the influence of ethanol-induced dehydration and genipin crosslinking on type I collagen scaffolds and compare their chemical and thermal properties with native murine Achilles tendons. Fourier Transform Infrared Spectroscopy (FTIR) demonstrated that ethanol treatment reduced molecularly bound water (from 127.3 to 48.0 a.u.) and caused shifts in amide bands (Amide I upshift, Amide II & III downshifts) commonly associated with dehydration, bringing values closer to those of native tendon. Genipin crosslinking further increased similarity to tendon, particularly in thermal behavior as measured by thermogravimetric analysis (TGA). Ten millimolar genipin-treated scaffolds pre-rehydration most closely approximated tendon but diverged post-rehydration. Crosslinking strongly restricted scaffold rehydration (final swelling factor 44.46 vs. 4.06) and altered mechanical properties. Together, these results demonstrate that dehydration and crosslinking influence collagen scaffolds across molecular, thermal, hydration, and mechanical properties. Careful control of these processing conditions, particularly hydration history, is therefore important for collagen-based in vitro models mimicking physicochemical features of native tendon.

## 1. Introduction

Although the use of native tissues and animal models remains the gold standard for investigating the properties and behaviors of musculoskeletal tissues, there is growing interest in developing synthetic systems and scaffolds that replicate key tissue behaviors as simplified in vitro models. Tendons, almost entirely comprising type I collagen, lend themselves to synthetic scaffold-based models in research [1,2,3]. The goal of these scaffolds is to replicate the composition, structure, and mechanical properties of native tissue while eliminating the variability of cadaver tissue or the complexity of animal models.

Tendon structure is characterized by aligned hydrated collagen fibers that are highly crosslinked, resulting in an extremely strong matrix that is particularly tough under tension [4]. In vivo crosslinking is both enzymatic and non-enzymatic, comprised mainly of pyridinoline links and advanced glycation end products (AGE’s) respectively [5,6,7,8,9,10]. The accumulation of these crosslinks alters collagen spacing and movement, significantly affecting the tendon mechanics. However, it also has significant chemical, thermal and biological effects. Increased crosslinking dehydrates the tendon, eliminating up to 30% of the initial water content and changing the mechanical stress environment [11,12]. This leads to significant differences in the tendon’s thermal stability [11]. In addition, these chemical changes significantly affect the cellular response of tendon cells which are reactive to collagen chemistry, hydration, and mechanical environments [13,14]. Therefore, it is not only necessary to make tendon-like collagen scaffolds that mimic the tissue density and mechanics, but to also consider the thermal and chemical similarities between artificial systems and native tendon.

Many studies have sought to create high-density aligned collagen systems for tendon replacement, tissue engineering, or modeling [15,16,17,18]. These approaches seek to reproduce tendon-like mechanical properties and promote tenocyte differentiation and activity. However, nearly all these studies use ethanol, cyclic dehydration, or crosslinking treatments on their scaffolds without investigating the effects of these treatments on the scaffold chemical and thermal behavior. Our previous work has indicated that ethanol post-treatment had significant effects on scaffold structure and mechanical response, specifically resulting in a loss of both bulk and molecular bound water, and an increase in stiffness [19]. Artificial crosslinking also promotes dehydration and changes in thermal stability [11]. There are several methods of crosslinking collagen in vitro, but to maintain similarity to tendons, a chemical crosslinking method was chosen. Physical crosslinking, while still common, can result in crosslinks that are temporary [20,21], which may be desirable for specific applications but is not ideal for a tendon scaffold model. The two most common chemical crosslinking methods are glutaraldehyde [22,23,24] and genipin (GP) [25,26,27]. Glutaraldehyde, however, has been shown to disrupt the collagen structure and increase the toxicity of the scaffold [28,29]. For these reasons, this study used genipin as the chosen crosslinking method. The chemistry of the scaffolds is affected by cyclic dehydration and rehydration [19]. While these effects have been investigated independently, comparisons of the resulting scaffolds to native tendon have not been examined. Therefore, in this study, we seek to elucidate the effects of ethanol-induced dehydration combined with crosslinking on collagen matrices and compare them to standard murine tendons. This will allow us to identify the treatments that create collagen scaffolds most chemically and thermally similar to native tendon.

## 2. Results and Discussion

### 2.1. Effects of Ethanol Treatment, Genipin Crosslinking, and Rehydration on Collagen Structure

Molecular structure was examined by FTIR, specifically Amides I, II, and III peak center and full-width-half-max. The broad series of peaks between 2500 and 3750 cm^−1^, representative of O-H bond stretching, is considered representative of molecular bound water and is plotted as such. For tendon samples, the peaks identified as lipids, specifically at 1745 cm^−1^, and 2860 and 2935 cm^−1^, were fit with adjacent collagen peaks and subtracted to get a signal comparable to collagen alone (Figure 1a). Tendon samples had a significantly lower water peak area, indicating less bound molecular water, which was comparable to the decrease seen with ethanol treatment (Figure 1b). An “upshift” refers to an increase in peak center location, and a “downshift” to a decrease in peak center location. With Amide I there was a trending upshift with ethanol, a significant increase with tendon, as well as a trending increase in FWHM (Figure 1c,d). The increased width indicates increased disorder. Amides II and III had a reversed trend, with downshifts occurring in both ethanol and tendon samples, although both had increases in FWHM (Figure 1e–h). This pattern, an upshift in Amide I and downshifts in Amides II and III, although not always statistically significant, is commonly associated with decreased hydration [19,30].

Crosslinking with genipin at either 10 or 25 mM did not change the bound molecular water or cause any statistically significant changes in FTIR structural measures pre-rehydration compared to ethanol-treated scaffolds (Figure 2a). Rehydration and subsequent air drying, however, did reveal changes with genipin. Molecular water was lost in all groups, not dependent on crosslinked state, as was the upshift seen in Amide I (Figure 2b). The changes in Amides II and III, however, did show increased downshifts with increased genipin concentration (Figure 2c–h). Dashed lines on all graphs indicate the value obtained from tendons for reference. Rehydration caused significant downshifts in all groups, resulting in values further away from the tendon reference. This indicates that although genipin crosslinking resulted in scaffolds more like tendon pre-rehydration, the rehydration process pushed the scaffolds further away from tendon reference values.

### 2.2. Thermal Stability of Crosslinked Scaffolds and Murine Tendons

Thermal stability was analyzed via TGA. There were two notable points where mass was lost, specifically around 140 °C and between 200 and 500 °C. This first loss is the loss of any remaining water present in the scaffolds and is less in both tendon samples and crosslinked samples, which correlates with the lost molecular water seen in FTIR. The second period of mass loss, between 200 and 500 °C, is associated with collagen thermal degradation. An increased amount of mass lost during this degradation is due to decreased thermal stability of the collagen scaffolds. Ethanol slightly increased this instability (Figure 3a), but 10 mM genipin crosslinking even more so, resulting in values very similar to those of tendons (Figure 3c–e). There was a decrease in initial mass loss with both crosslinking and tendons, which does correlate with molecular water loss (Figure 3c). This is also indicated by the resulting mass at 500 °C. In both mass lost during collagen degradation and mass remaining at 500 °C, 10 mM genipin crosslinked scaffolds were the most like tendons (Figure 3d,e).

### 2.3. Mechanical Properties Under Tension of Genipin Crosslinked Collagen Scaffolds

Crosslinking the scaffolds with genipin completely restricted any rehydration capacity at both 10 and 25 mM concentrations (Figure 4a). While ethanol-only scaffolds reached a swelling factor of almost 60 after 10 days, the crosslinked scaffolds swelled to approximately four times their dry volume within 1 h and then remained in this state. This lack of bulk water in the scaffolds naturally resulted in significantly increased strength post-rehydration, as well as decreased ultimate strain (Figure 4b–d). The only significant change pre-rehydration was a dramatic decrease in post-failure energy, essentially indicating a loss of plastic deformation after the initial failure point (Figure 4f). The crosslinked scaffolds were significantly stronger and tougher scaffolds post-rehydration due to their lack of swelling and bulk water. This again indicates that the mechanical response of scaffolds is almost completely determined by bulk water content.

### 2.4. Discussion

This study demonstrates that common processing techniques for the formation of collagen scaffolds significantly alter collagen hydration, molecular structure, thermal behavior, and mechanical properties. Ethanol-induced dehydration shifted several FTIR measures toward those observed in native murine tendon, while genipin crosslinking further increased similarities in thermal behavior. However, these treatments also altered scaffold rehydration and mechanical response, demonstrating that processing methods used to improve tendon-like scaffold properties can have coupled effects across multiple structural and functional scales.

Ethanol treatment produced the first major shift in scaffold properties towards those of native tendon. Historically, ethanol treatment of scaffolds has been a common step in manufacture and processing and has been reported to reduce both bulk water and tightly bound molecular water affecting scaffold structure and behavior [19,30]. Here, FTIR demonstrated decreased molecularly bound water accompanied by shifts in the Amide I, II, and III bands, consistent with changes previously associated with collagen dehydration (Figure 1). These changes are particularly relevant when comparing low-density collagen scaffolds with native tendon, where collagen fibrils are densely packed and the available space for bulk and molecularly associated water is substantially reduced. These changes were seen not only in FTIR, but also in TGA, where both ethanol-treated scaffolds and tendons had increased mass loss between 200 and 500 °C as compared to the untreated scaffolds, suggesting decreased thermal stability (Figure 3). It is important to note that while the loss is assumed to be collagen degradation, tendons do include other proteins and biological compounds which also affect stability. Thus, it appears that the dehydration induced by EtOH treatment increases the similarity between the scaffolds and the tendon in terms of structural chemistry and thermal properties.

Genipin crosslinking produced additional changes beyond those induced by ethanol treatment. Tendons are highly crosslinked, and while it is difficult to replicate these conditions in a benchtop setting, genipin crosslinking has been used as a natural method that does not involve denaturing chemicals or processes [25,26,32,33,34,35]. In this study there was little difference between 10 and 25 mM genipin concentrations, though both were more like tendons than uncrosslinked samples (Figure S1b). The largest difference seen with crosslinking was the complete lack of rehydration capacity (Figure 4). This is unsurprising; the crosslinks present will hold the scaffold in place and prevent expansion to accommodate water, resulting in a smaller and therefore much stronger scaffold with a higher density of collagen. Previous studies have also shown that the tensile response of collagen scaffolds is largely determined by bulk water content, and so a decreased water content does result in increased strength [19]. The other effect of crosslinking was a loss of post-failure behavior. This is likely due to the crosslinks restricting sliding and rearrangement of the collagen fibrils and fibers during testing, meaning that once the failure point is reached, the scaffold can no longer deform to extend this plastic region.

Importantly, rehydration did not simply reverse the structural effects of dehydration. The rehydration process resulted in significant loss of molecular water, and although this value was the closest to that of tendons, the rehydration process also caused other structural changes, such as a further upshift in Amide I and downshifts in Amides II and III, which deviated from the tendon reference (Figure 2). Consequently, scaffold hydration history should be considered when comparing collagen-based systems or designing processing protocols intended to reproduce native tissue properties.

Principal component analysis (Figure S1) served to summarize the similarities between tendons and scaffolds across several methods. By combining several measures, the analysis suggests that ethanol treatment causes initial shifts towards tendon, and the addition of genipin crosslinking results in the most comparable scaffolds to murine tendons. Since the mechanical testing system used in this study would not have been applicable to tendons, it was not possible to include mechanical testing results in the principal component analysis, limiting its accuracy.

Major differences between tendons and the scaffolds used here can be attributed to the significant differences in collagen density. Murine tendons have tightly packed collagen with a high spatial density [36], which is not reproducible in this model due to the limitations of collagen solubility. Crosslinking in vivo is also dramatically different from genipin crosslinking. While they both form irreversible covalent bonds between collagen molecules, it has not yet been possible to truly replicate in vivo crosslinking patterns in vitro. Future studies incorporating higher-density aligned collagen systems and direct mechanical comparison with native tendon will be necessary to evaluate functional similarity more completely.

## 3. Conclusions

Overall, these results demonstrate that ethanol-induced dehydration and genipin crosslinking substantially alter the physicochemical and mechanical properties of type I collagen scaffolds. Ethanol treatment reproduced several hydration-associated molecular features observed in native tendon, while genipin crosslinking further modified thermal behavior and strongly restricted subsequent water uptake. Together, these findings highlight hydration and crosslinking as interconnected determinants of collagen scaffold behavior and demonstrate that processing history must be considered when developing collagen-based in vitro models of tendon. Rather than reproducing all features of native tendon, these scaffolds provide a simplified and tunable system for investigating relationships among collagen structure, hydration, and mechanical behavior.

## 4. Materials and Methods

### 4.1. Collagen Scaffold Manufacture

Collagen scaffolds were made from acid-soluble lyophilized bovine type I collagen (calf skin, Elastin Products Company, Owensville, MO, USA) as previously described [19,37]. Collagen, 10 mg/mL, was dissolved in cold 5 mM hydrochloric acid, pulled into 1 mL syringes and tapped to remove bubbles. Syringes were left at 4 °C for 24–48 h until all bubbles had been removed. Collagen was injected into custom 3D printed molds and immersed in a polymerizing buffer consisting of 30 mM N-tris(dihydroxy methyl)methyl-2-aminoethane sulfonic acid, 30 mM sodium dihydrophosphate, and 135 mM sodium chloride, adjusted to pH 7.5 by gradual addition of 1 M sodium hydroxide. Molds were either shaped as small disks or hoop shapes with an outer diameter 13 mm, inner diameter 8 mm, and height 11 mm. Scaffolds were made as disk shapes for thermogravimetric analysis to keep the scaffolds within weight limits. Molds were submerged for 90 min at 30 °C, and scaffolds carefully removed and stored in deionized water (DiH_2_O). In this study, collagen fibers were not aligned in the scaffold due to the challenges associated with fiber alignment in a circular mold, chosen for mechanical testing reasons, where it is not possible to align fibers with an electric field or weave them into a specific structure, for example [38,39,40,41]. This was considered acceptable as the focus of this study was determining the chemical and thermal properties of the scaffolds.

Genipin is a compound originally derived from the fruit of the *Gardenia jasminoides* plant, has been shown to effectively crosslink collagen across multiple applications, and is biocompatible and non-toxic [32,33,42,43]. Ten millimolar is considered a high concentration of genipin [32,44]. Due to tendon’s highly crosslinked nature, we investigated two concentrations: 10 mM and 25 mM. In our early experiments, both concentrations resulted in alterations of the collagen structure and mechanical properties compared to non-crosslinked samples but did not show significant differences between the two. This suggested that at 10 mM the scaffold had reached some threshold of crosslinking, and additional genipin could not add more crosslinks beyond this level. Crosslinking was performed by dissolving genipin (Thermo Scientific cat. 466640010, Waltham, MA, USA) in DiH_2_O (10 mM or 25 mM) and submerging the scaffolds in the solution for 24 h. Scaffolds turned blue when crosslinked. Scaffolds were subsequently rinsed in deionized water to remove excess genipin.

Ethanol post-treatment was performed after crosslinking and involved sequential treatment of scaffolds with increasing concentrations of ethanol (70%, 95%, 100%) each for 1 h. Scaffolds were left in 100% ethanol overnight to ensure complete saturation.

Disk scaffolds were air-dried flat, and hoop scaffolds were mounted on 1000 μL pipette tips to maintain their shape for further analysis.

### 4.2. Tendon Isolation

Murine Achilles tendons were isolated from 6-month-old male C57BL/6 mouse carcasses. Animals were obtained from a control group of a previous study via shared tissue collection (Protocol number: AP-201030-0826). Hind limbs from 6 animals were dissected, Achilles tendons were cleanly isolated, removing as much muscle and adipose tissue as possible. Both left and right tendons were used in this study. Tendons were air-dried flat for analysis. Tendons were approximately 4 mm in length, 2 mm in width, and less than 1 mm in thickness.

### 4.3. Fourier Transform Infrared Spectroscopy (FTIR)

Attenuated Total Reflectance (ATR) FTIR was performed as previously described [19]. Briefly, dry samples were scanned with their own background measurements and 16 scans summed for each spectrum (Thermo Fisher Nicolet iS20, Waltham, MA, USA). Peak fitting was performed in OriginPro (OriginPro 2021b, OriginLab Corporation, Northampton, MA, USA). Straight line background was subtracted from the region of interest and approximate peak locations specified. A Gaussian model was fit to a tolerance of 10^−6^, with an r^2^ value of greater than 0.99. Peak locations, areas, and full-width-half-max (FWHM) were extracted for each model fit. Peaks chosen to fit were based on prior experience and literature [45,46,47,48,49,50,51].

### 4.4. Thermogravimetric Analysis (TGA)

Thermogravimetric analysis was performed on dry samples (TA Instruments, TGA Q500-0188, New Castle, DE, USA). Samples were burned in platinum pans in pure nitrogen from room temperature up to 800 °C. Loss of mass curves as a function of temperature were analyzed in a custom MATLAB (R2026a) script which calculated inflection points and percentage of mass lost between these points. Visualization was performed in TGA software (TA Universal Analysis, New Castle, DE, USA).

### 4.5. Mechanical Testing

Hoop scaffolds were mechanically tested under tension to failure in a hydrated state (either initial hydration or dried and subsequently rehydrated). Murine tendons were not mechanically tested as much previous literature exists on the mechanical properties of murine tendons. Hoop scaffolds were mounted on custom T-shaped rigs and tested as previously described (TA Electroforce ELF3200, Electroforce load cell, Eden Prairie, MN, USA) [19]. Briefly, scaffolds were held at zero strain for 2 s and then pulled at 0.5 mm/s until failure. A custom MATLAB script calculated stress, strain, ultimate failure, material toughness, resilience, and Young’s modulus.

### 4.6. Rehydration and Swelling Experiments

Scaffold mass was used to calculate swelling factors under various conditions. To obtain mass measurements, scaffolds were removed from solution and dried carefully using a Kim wipe to remove excess surface liquid before weighing. Since scaffolds used in this study were 100% bovine collagen type I, swelling factor was calculated using Equation (1), accounting for the known density of type I collagen (1.32 g/cm^3^) [19,52].

(1) $$Swelling\;Factor\;\left(SF\right)=\left(1.32\times \frac{M_{wet\;col1}}{M_{dry\;col1}}\right)-0.32$$

where *M_wet col_*_1_ = mass of collagen with water or ethanol and *M_dry col_*_1_ = mass of dry collagen [52,53]. Dry weights of scaffolds were obtained after air drying; for rehydration experiments, this was after the final timepoint.

Rehydration experiments were performed by immersing air-dried scaffolds in deionized water for up to 10 days. Mass measurements to calculate swelling were performed as above with Equation (1). Since some techniques required dry samples (TGA, FTIR, Raman), scaffolds were air-dried after rehydration for analysis.

### 4.7. Principal Component Analysis

Principal component analysis and correlation analyses were performed to establish relationships between different technical measurements and identify trends within treatment groups. Principal component analysis looked at ethanol dehydration and crosslinking as the core variables, so only 10 mM genipin was included. PCA was based on FTIR and TGA data. A separate correlation analysis was performed to include mechanical testing information and swelling factors.

### 4.8. Statistical Analysis

All statistical analyses were performed in GraphPad Prism (v.10). Data was analyzed with *t*-tests and one- or two-way ANOVAs were performed as appropriate. Statistical significance was defined as *p* < 0.05. Plots show mean values and standard deviations.

## Figures and Tables

**Figure 1:**
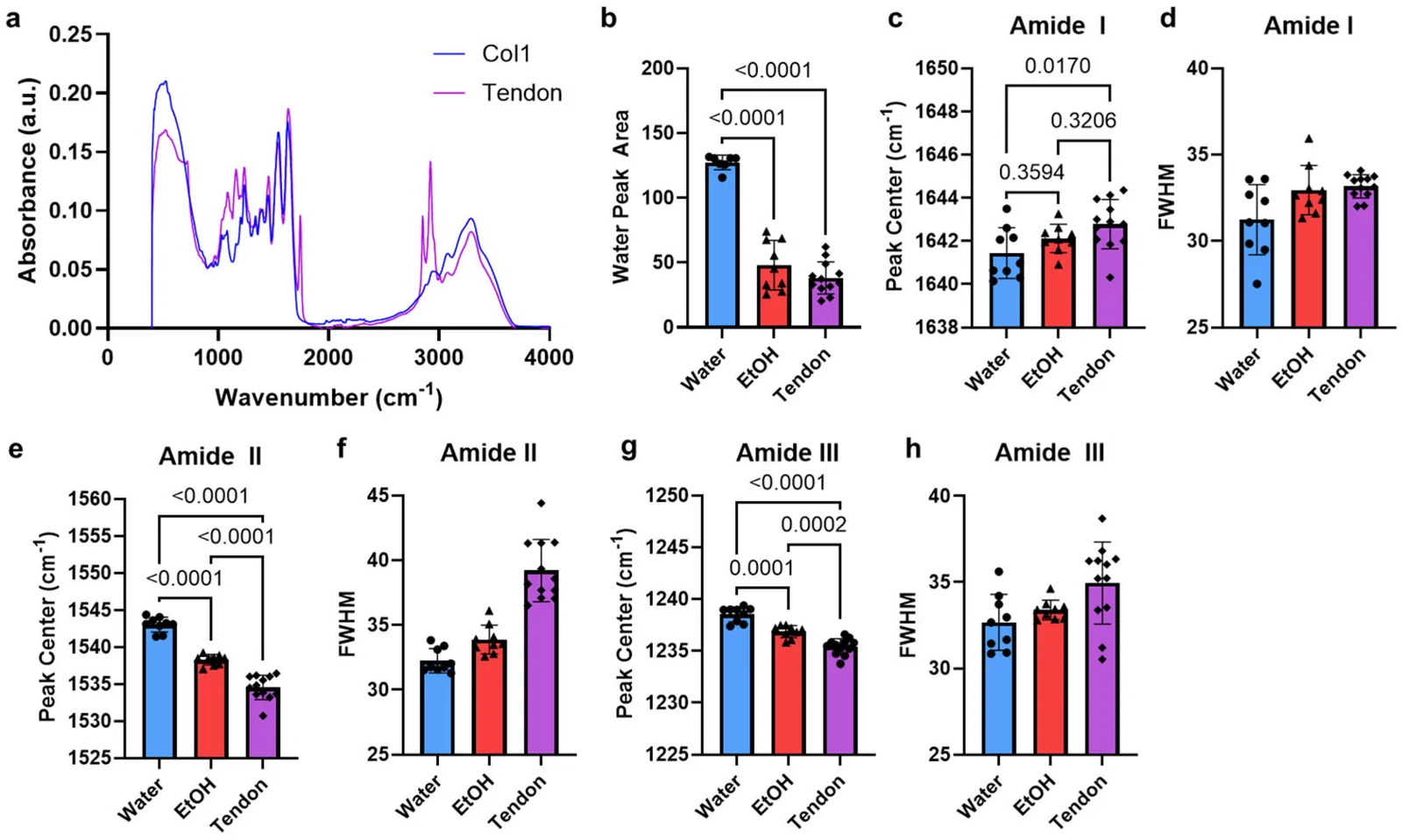
Fourier Transfer Infrared Spectroscopy (FTIR) results for collagen scaffolds and tendons. (**a**) Representative spectra for collagen scaffolds and murine tendons. (**b**) Total water peak area between 2500 and 3750 cm^−1^ for untreated collagen scaffolds, ethanol treated scaffolds, and murine tendons. (**c**) Amide I peak center. (**d**) Amide I full-width-half-max. (**e**) Amide II peak center. (**f**) Amide II FWHM. (**g**) Amide III peak center. (**h**) Amide III FWHM.

**Figure 2:**
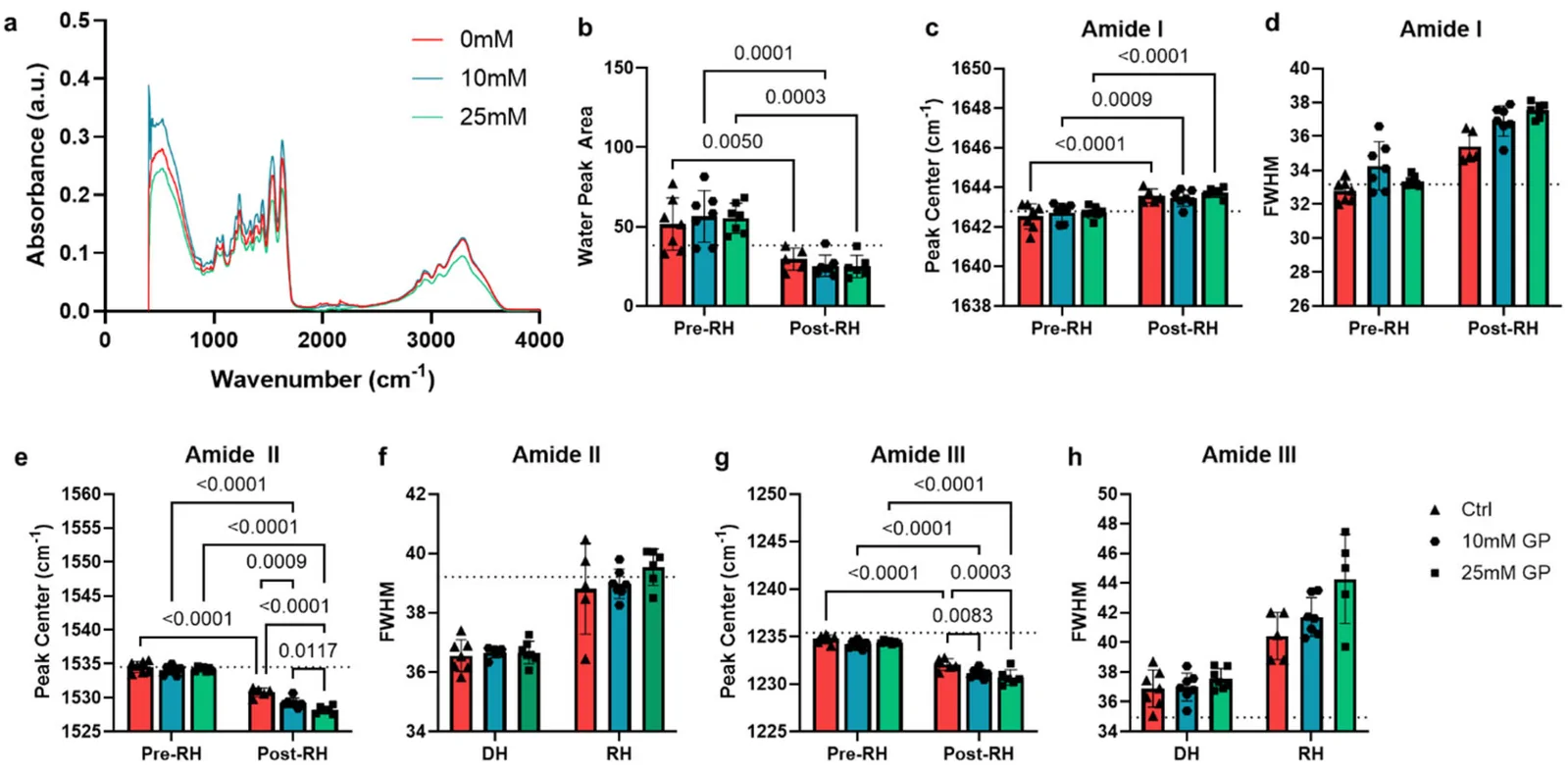
Fourier Transform Infrared Spectroscopy (FTIR) results for crosslinked collagen scaffolds. Dashed lines indicate average values for murine tendons for reference. (**a**) Representative spectra for control, 10 mM, and 25 mM genipin crosslinked scaffolds. (**b**) Total water peak area pre- and post-rehydration. (**c**) Amide I peak center. (**d**) Amide II FWHM. (**e**) Amide II peak center. (**f**) Amide II FWHM. (**g**) Amide III peak center. (**h**) Amide III FWHM.

**Figure 3:**
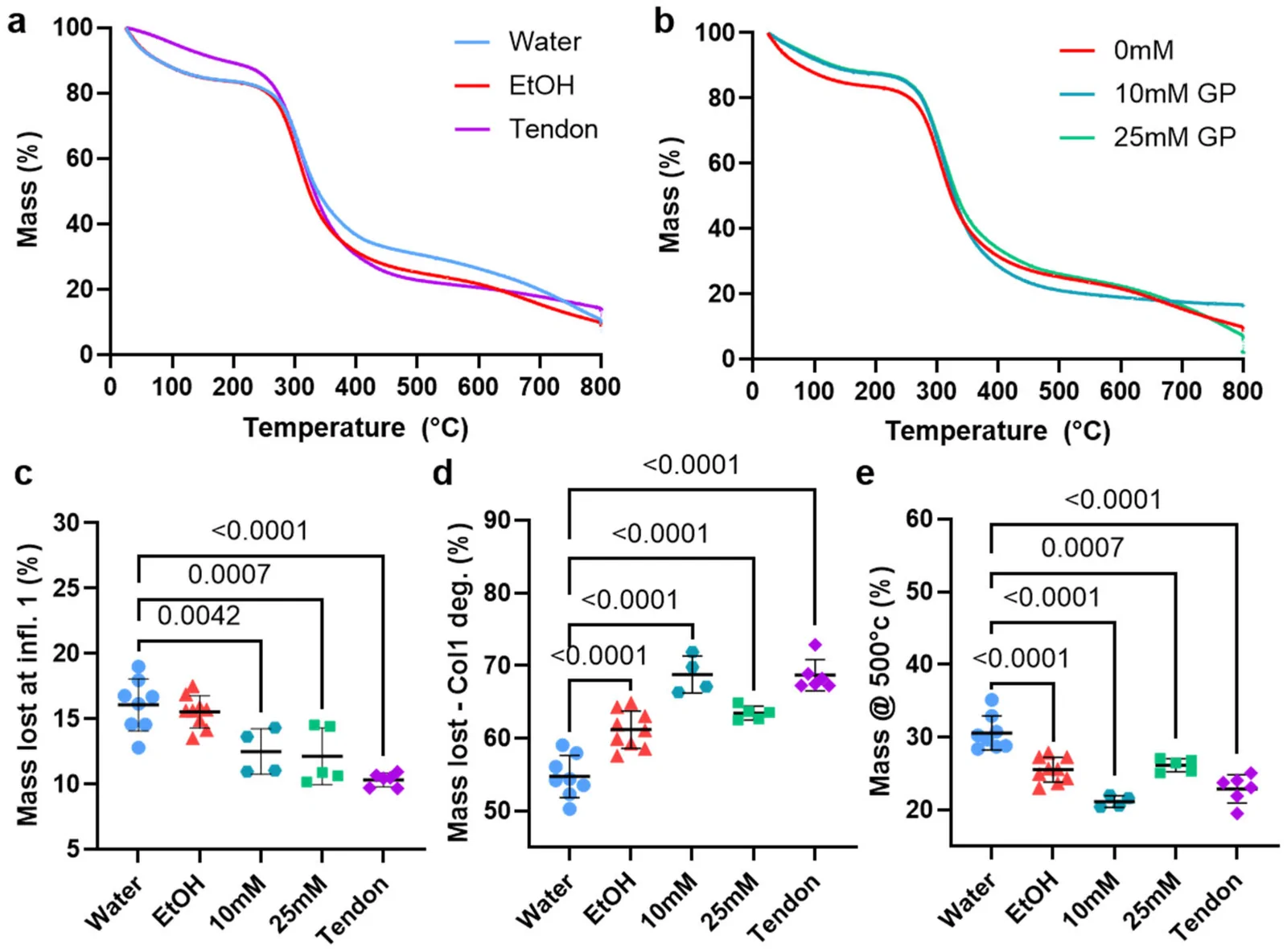
Thermogravimetric analysis (TGA) results for collagen scaffolds and murine tendons. (**a**) Average thermal degradation curves for non-ethanol-treated, ethanol-treated, and murine tendons. (**b**) Average thermal degradation curves for control, 10 mM, and 25 mM genipin crosslinked scaffolds. (**c**) Mass lost by first inflection point, at approximately 140 °C. (**d**) Mass lost during collagen degradation drop, approximately 200–500 °C. (**e**) Mass remaining at 500 °C.

**Figure 4:**
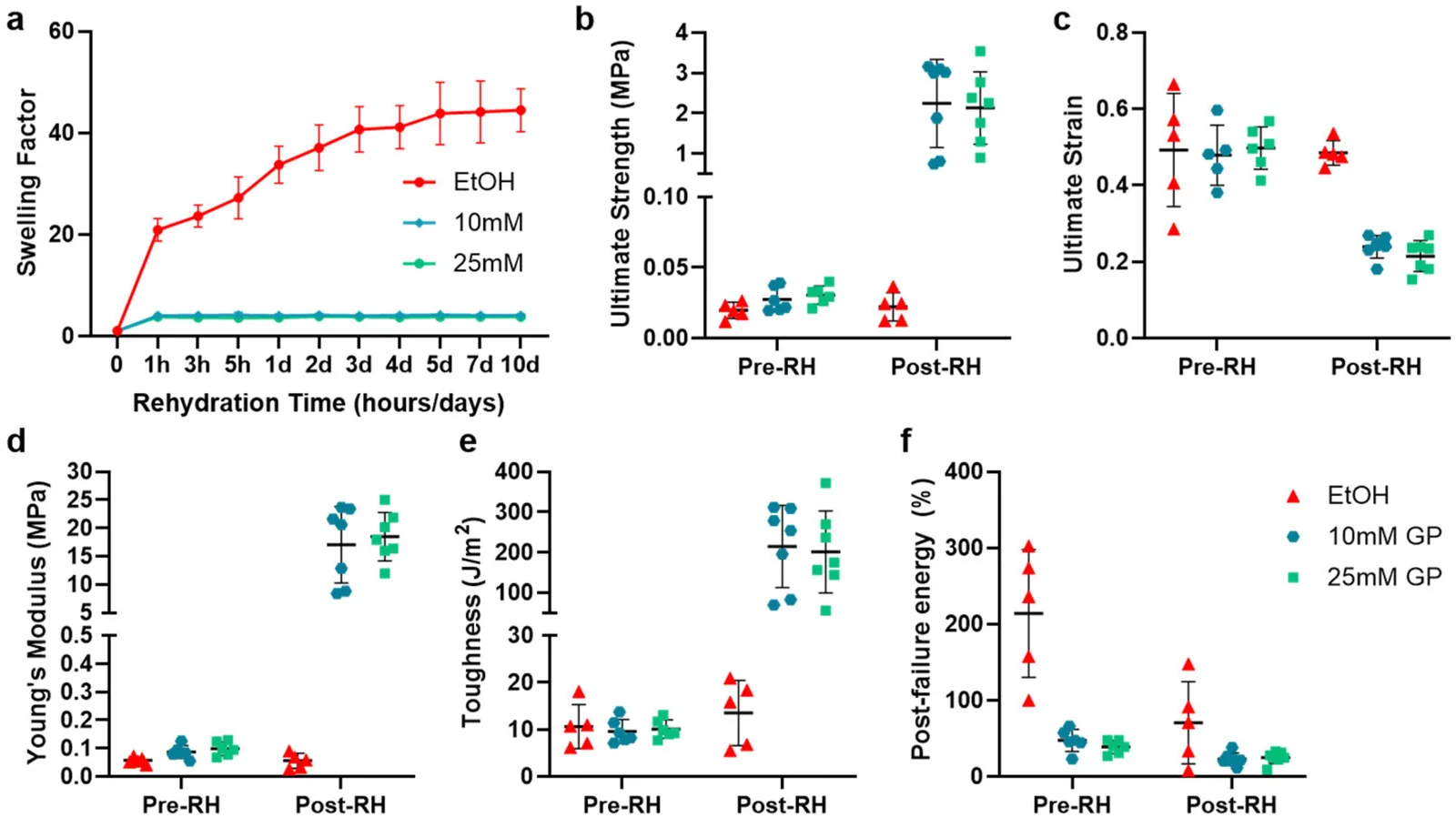
Rehydration swelling and tensile test results for crosslinked scaffolds. (**a**) Volumetric swelling factor over 10 days of rehydration. Ten and 25 mM genipin crosslinked scaffolds overlap. (**b**) Ultimate strength of crosslinked scaffolds pre- and post-rehydration. Murine tendon ≈ 20 MPa [31]. (**c**) Ultimate strain of crosslinked scaffolds pre- and post-rehydration. Murine tendon ≈ 0.2 [31]. (**d**) Young’s modulus of crosslinked scaffolds pre- and post-rehydration. Murine tendon ≈ 150 MPa [31]. (**e**) Material toughness of crosslinked scaffolds pre- and post-rehydration. (**f**) Post-failure energy of crosslinked scaffolds pre- and post-rehydration.

## Data Availability

All data available upon reasonable request.

## Supplementary Materials

The following supporting information can be downloaded at https://www.mdpi.com/article/10.3390/gels12100871/s1, Figure S1: Correlation and principle component analysis between groups.

## Abbreviations

The following abbreviations are used in this manuscript:

FTIR	Fourier Transform Infrared Spectroscopy
TGA	thermogravimetric analysis
FWHM	full-width-half-maximum
mM	millimolar
